# A 14-Day Double-Blind, Randomized, Controlled Crossover Intervention Study with Anti-Bacterial Benzyl Isothiocyanate from Nasturtium (*Tropaeolum majus*) on Human Gut Microbiome and Host Defense

**DOI:** 10.3390/nu16030373

**Published:** 2024-01-26

**Authors:** Simon P. Pfäffle, Corinna Herz, Eva Brombacher, Michele Proietti, Michael Gigl, Christoph K. Hofstetter, Verena K. Mittermeier-Kleßinger, Sophie Claßen, Hoai T. T. Tran, Dhairya Rajguru, Corinna Dawid, Clemens Kreutz, Stefan Günther, Evelyn Lamy

**Affiliations:** 1 Molecular Preventive Medicine, University Medical Center and Faculty of Medicine, University of Freiburg, Engesserstrasse 4, D-79108 Freiburg, Germany; 2 Institute of Pharmaceutical Sciences, Faculty of Chemistry and Pharmacy, University of Freiburg, Hermann-Herder-Strasse 9, D-79104 Freiburg, Germany; 3 Institute of Medical Biometry and Statistics, University Medical Center and Faculty of Medicine, University of Freiburg, Stefan-Meier-Str. 26, D-79104 Freiburg, Germany; 4 Faculty of Biology, University of Freiburg, Schänzlestr. 1, D-79104 Freiburg, Germany; 5 Spemann Graduate School of Biology and Medicine (SGBM), University of Freiburg, Albertstr. 19A, D-79104 Freiburg, Germany; 6 Centre for Integrative Biological Signaling Studies (CIBSS), University of Freiburg, Schänzlestr. 18, D-79104 Freiburg, Germany; 7 Center for Chronic Immunodeficiency (CCI), Microbiome Core Facility, Breisacher Strasse 115, D-79106 Freiburg, Germany; 8 Chair of Food Chemistry and Molecular Sensory Science, TUM School of Life Sciences, Technical University of Munich, Lise-Meitner-Strasse 34, D-85354 Freising, Germany; 9 Bavarian Center for Biomolecular Mass Spectrometry, TUM School of Life Sciences, Technical University of Munich, Gregor-Mendel-Strasse 4, D-85354 Freising, Germany; 10 Institute of Molecular Medicine and Cell Research, Faculty of Medicine, University of Freiburg, D-79104 Freiburg, Germany

**Keywords:** nasturtium, BITC, gut microbiome, antimicrobial, metabolome, *Escherichia coli*, human beta defensin 1

## Abstract

Despite substantial heterogeneity of studies, there is evidence that antibiotics commonly used in primary care influence the composition of the gastrointestinal microbiota in terms of changing their composition and/or diversity. Benzyl isothiocyanate (BITC) from the food and medicinal plant nasturtium (*Tropaeolum majus*) is known for its antimicrobial activity and is used for the treatment of infections of the draining urinary tract and upper respiratory tract. Against this background, we raised the question of whether a 14 d nasturtium intervention (3 g daily, N = 30 healthy females) could also impact the normal gut microbiota composition. Spot urinary BITC excretion highly correlated with a weak but significant antibacterial effect against *Escherichia coli*. A significant increase in human beta defensin 1 as a parameter for host defense was seen in urine and exhaled breath condensate (EBC) upon verum intervention. Pre-to-post analysis revealed that mean gut microbiome composition did not significantly differ between groups, nor did the circulating serum metabolome. On an individual level, some large changes were observed between sampling points, however. Explorative Spearman rank correlation analysis in subgroups revealed associations between gut microbiota and the circulating metabolome, as well as between changes in blood markers and bacterial gut species.

## 1. Introduction

Antibiotics are commonly used in the treatment of bacterial infections. Even though these medications are saving millions of lives, the use of antibiotics is also increasingly leading to multidrug-resistant microorganisms. New antibiotics are lacking, and thus there has been great interest in plants’ antimicrobial properties [1,2,3].

These plant-derived antimicrobial compounds are present in diverse plant components, encompassing roots, stems, leaves, flowers, fruits, and seeds. Nasturtium (Indian cress, *Tropaeolum majus,* order Brassicales, family Tropaeolaceae) is one such plant known for its medicinal properties and compliance with the German “Commission E” standards for herbal medicinal products established in 1978 [4]. These standards aim to ensure the quality and therapeutic effectiveness of herbal remedies. Nasturtium is known for its anti-bacterial, anti-fungal, and anti-viral potential. It is widely used in cooking and as a dietary supplement; in combination with horseradish, it is used for the treatment of infections of the draining urinary tract and upper respiratory tract [5,6], and is also recommended for the prevention of recurrent urinary tract infections (UTIs) as per the S3 guideline [5].

Among the most important phytochemicals produced by Brassicales plants are the biologically inactive pro-drugs called glucosinolates (GLSs). The common structure of GLSs comprises a *β*-D-thioglucose group, a sulfonated oxime moiety, and a variable side-chain derived from amino acids [7]. Upon cell injury, GLSs come into contact with the plants’ enzyme myrosinase, a heat-sensitive thioglucosidase, which cleaves the sugar moiety and leads to the formation of isothiocyanates (ITCs), thiocyanates, or nitriles [8]. ITCs are very reactive products and have been intensively investigated for their health-promoting and therapeutic activity [9,10,11]. The degradation product of glucotropaeolin, benzyl isothiocyanate (BITC), is thought to be mainly responsible for the antimicrobial activity observed in nasturtium [9]. BITC was found to inhibit the growth of different Gram-positive and Gram-negative bacteria, which were isolated from human fecal material [8]. This raises the prospect of controlling human pathogens through dietary intervention. One mechanistic explanation of the antimicrobial activity is based on the reaction of ITC with thiol groups to form dithiocarbamates, as well as with amino groups to form thioureases [12,13]. These reactions could lead to increased oxidation and inhibition of vital proteins and/or enzymes, resulting in bacterial cell death. In addition, using the Gram-negative *Campylobacter jejuni* NCTC1118i68, transcriptomic analysis suggested that BITC triggers bacterial signaling pathways, inducing heat shock and oxidative stress response, protein aggregation, and the dysfunction of energy metabolism, leading to bacterial death [14].

On the other hand, this antimicrobial capacity of BITC/nasturtium might also imply the potential for adverse consequences for the homeostasis of the healthy gut microbiome.

Today, various antibiotics have been recognized as major disruptors of the gut microbiota, leading to reduced diversity and dysbiosis, which are associated in consequence with adverse health effects [15,16,17,18]. Even a single antibiotic treatment was found to trigger considerable microbial shifts and antibiotic resistance enrichment in the feces of healthy humans [16]. Thus, the aim of the present study was to investigate the effect of BITC-containing nasturtium on the bacterial microbiome in the human gut of healthy subjects. This was investigated as an exploratory endpoint in the context of a two-week randomized, double-blind, controlled crossover nasturtium food intervention.

## 2. Materials and Methods

### 2.1. Study Design

This study is a follow-up to a previously conducted trial registered on the German Clinical Trials Register (DRKS) with the ID DRKS00016548. Details of the design and results of the primary study, which assessed the impact of BITC-containing nasturtium on the lipid regulator prostaglandin E2 in human blood samples, have been already reported in [19]. In the present study, we focus on the analysis of secondary parameters. Briefly, the study was conducted as a double-blind, monocentric, controlled, randomized crossover intervention. Subjects were all female, between 20 and 45 years of age, healthy, and non-smokers. Exclusion criteria were acute and chronic diseases, acute gastric/intestinal ulcers, acute kidney inflammation, pregnancy, lactation, intake of antibiotics (acute, during the last three months), known allergy to cruciferous vegetables, or a BMI > 25 and <18.5. Following the protocol, 1.5 g nasturtium plant powder (standardized dried powder of *T. majus* flowers and leaves, provided by Repha GmbH, Langenhagen, Germany) was freshly mixed with 20 mL of water and consumed by subjects two times per day, which over the 14-day intervention period corresponded to a total intake of 42 g. Verum contained nasturtium with preformed BITC in the amount of 18.87 ± 4.5 µmol, while the amount of BITC in nasturtium used as control was almost negligible (0.07 ± 0.06 µmol). BITC content was analyzed using GC-MS and is described in detail elsewhere [19]. The subjects were asked to follow their usual dietary habits but excluding foods rich in GLS. In addition to other tests, stool samples were collected from N = 34 subjects at the beginning and end of each intervention phase. No DNA could be isolated from stool samples of 4 subjects due to the low amount. High-throughput 16S ribosomal RNA (rRNA) gene sequencing was performed on N = 30. In the first intervention phase, subjects were randomly assigned to either verum or control, each of which was administered for two weeks. Following a crossover procedure, in the second intervention phase the subjects who had started with the verum sample received the control and vice versa. In between, a wash-out phase of at least two weeks was used to exclude possible carry-over effects. The treatment order was randomized by dividing participants using a random-number generator. Participants documented their diet, medications, unusual exercise, and general well-being on a daily basis.

### 2.2. Collection of Stool, Urine, and Serum Samples

Subjects were asked to collect stool samples before the first consumption of nasturtium on day 1 and on the last intake on day 14 using the OMNIgene-GUT Microbiome kit (DNA Genotek, Ottawa, ON, Canada). Aliquots of stool samples were stored at −80 °C until analysis. Blood samples were collected in the morning before the first intake of nasturtium and 4 h after the last intake on day 14 in coagulant vacutainers via venipuncture. The tubes were gently shaken after blood collection and centrifuged at 2000× *g* for 10 min at 4 °C. The upper layer (serum) was collected in 0.6 mL frozen tubes, overlayed with nitrogen, and stored at −80 °C until further analysis. On day 1 before taking nasturtium, and on day 14 before the last nasturtium intake, the subjects were asked to collect a spontaneous urine sample and store it in the fridge. When subjects arrived at the study center on day 1 or 14, they provided us with the urine samples, which were then aliquoted and stored at −80 °C until analysis.

### 2.3. Collection of Exhaled Breath Condensate (EBC)

Exhaled breath condensate (EBC) was collected in the morning before the first intake of nasturtium and 4 h after the last intake on day 14. Participants were asked to breathe uniformly for 10 min through the mouth into a Teflon-lined tube (diameter 12 mm, Carl Roth GmbH & Co. KG, Karlsruhe, Germany), cooled with dry ice as described in [20]. The collected EBC was transferred to a 5 mL reaction tube, snap frozen, and stored at −80 °C. For analysis, the samples were freeze dried (Alpha 2–4 LD, Martin Christ, Osterode am Harz, Germany), and the lyophilized samples were resuspended in 210 µL PBS w/o Ca^2+^ and Mg^2+^ (Life Technologies GmbH, Darmstadt, Germany). Protein quantity was assessed using a NanoDrop ND-1000 spectrophotometer at 230 nm (Thermo Scientific, Freiburg, Germany).

### 2.4. Faecal Microbial DNA Extraction

The fecal microbial DNA from stool samples was extracted using the QIAamp DNA Stool Mini Kit (Qiagen, Hilden, Germany), according to the manufacturer’s protocols. The DNA was quantified using a NanoDrop ND-1000 spectrophotometer (Thermo Scientific, Freiburg, Germany).

### 2.5. High-Throughput 16S Ribosomal RNA (rRNA) Gene Sequencing

The variable region 3 (V3) and 4 (V4) of the 16S rRNA gene was amplified using the forward primer 5′ TCGTCGGCAGCGTCAGATGTGTATAAGAGACAGCCTACGGGNGGCWGCAG and reverse primer 5′ GTCTCGTGGGCTCGGAGATGTGTATAAGAGACAGGACTACHVGGGTATCTAATCC and sequenced using the 16S sequencing library protocol provided by Illumina on an Illumina MiSeq System (Illumina, Inc., San Diego, CA, USA) as described elsewhere [21]. In addition, the ZymoBIOMICS Microbial Community DNA Standard from Zymo Research (Freiburg, Germany) was co-sequenced. One of the 30 datasets had to be excluded due to data incompleteness.

### 2.6. Bioinformatics Analysis

Sequence data were processed with the mothur software (v1.39.5) [22] implemented in the public workflow management server Galaxy (usegalaxy.eu, [23]). The analysis was carried out according to the MiSeq SOP as proposed by Kozich et al. [24]. Operational Taxonomic Units (OTUs) were picked against the Greengenes database (v13_8_99) [25] with at least 97% similarity. Very rare OTUs (less than three total counts) were excluded. The sequences were rarefied to a sampling depth of 34,894 sequences per sample. The associated taxonomy was visualized using the Phinch data visualization framework [26].

To estimate the functional gene profiles across genera, the PICRUSt package integrated in the mothur software was applied [27]. Subsequently, high-level phenotypes represented by KEGG pathways [28] were determined using the BugBase web application [29] with default settings (Sep-2021).

### 2.7. Metabolome Analysis

Targeted short chain fatty acid (SCFA) analysis was performed using a QTRAP 5500 triple quadrupole mass spectrometer (Sciex, Darmstadt, Germany) coupled to an ExionLC AD (Sciex, Darmstadt, Germany) ultrahigh performance liquid chromatography system, equipped with two ExionLC AD pump systems, an ExionLC degasser, an ExionLC AD autosampler, an ExionLC AD column oven, and an ExionLC controller. A multiple reaction monitoring (MRM) method was used for the detection and quantification of SCFA. The electrospray voltage was set to −4500 V, and the following MS parameters were used: curtain gas (35 psi), collision gas (medium), ion source gas 1 (55 psi), ion source gas 2 (65 psi), and temperature (500 °C). The parameters of the MRM were optimized using commercially available SCFA standards. Chromatographic separation was achieved on a 100 × 2.1 mm, 100 Å, 1.7 μm, Kinetex C18 column (Phenomenex, Aschaffenburg, Germany) using 0.1% formic acid as eluent A and 0.1% formic acid in acetonitrile as eluent B. An injection volume of 1 µL and a flow rate of 0.4 mL/min were used. The gradient elution started at 23% B, which was held for 3 min. Afterwards, the concentration was increased to 30% B within 1 min, then to 40% B within 2.5 min. Finally, the concentration was increased to 100% B within 1.5 min and held for an additional 1.5 min. This was followed by equilibration at starting conditions for 2.5 min. The column oven was set to 40 °C, and the autosampler to 15 °C. Data acquisition and instrumental control were performed with Analyst 1.7 software (Sciex, Darmstadt, Germany). Data evaluation was performed using MultiQuant (Sciex, Darmstadt, Germany). Serum samples (30 µL) were homogenized in methanol (270 µL) for 20 min at 10 °C using a Thermomix (Eppendorf, Hamburg, Germany). For SCFA analysis, the derivatization method of [30] was adapted, as previously reported [31]. Specifically, 40 µL of the sample extract was spiked with 15 µL of isotopically labeled standards (ca. 50 µM) and mixed with 20 µL 120 mM EDC HCl-6% pyridine-solution and 20 µL of 200 mM 3-NPH HCl solution. After shaking at 1000 rpm for 30 min at 40 °C using an Eppendorf Thermomix (Eppendorf, Hamburg, Germany), 900 µL acetonitrile/water (50/50, *v*/*v*) was added. After centrifugation at 11,337× *g* for 2 min, the clear supernatant was used for LC-MS/MS analysis.

Targeted analysis of amino acids and amino acid derivates was carried out as previously reported [32] utilizing a QTRAP 6500+ mass spectrometer (Sciex, Darmstadt, Germany) operated in positive ionization mode (ESI+), with an ion spray voltage of +5500 V. Nitrogen served as the curtain gas at 35 psi, while gas 1 and gas 2 were set at 55 psi and 65 psi, respectively. The source temperature was maintained at 450 °C. Multiple reaction monitoring (MRM) mode was utilized, with scheduled detection windows tailored to each metabolite. The UHPLC system (Sciex, Darmstadt, Germany) employed for chromatographic separation consisted of an ExionLC column oven AC, two ExionLC binary gradient pumps, an ExionLC system controller, and an ExionLC autosampler AD. The separation was carried out on an ACQUITY UPLC BEH Amide column (2.1 mm × 100 mm, 130 Å, 1.7 μm, Waters Corporation, Milford, Massachusetts). The column was maintained at a temperature of 40 °C. The mobile phase consisted of water (solvent A) and acetonitrile/water (95/5, *v*/*v*, solvent B), both containing 5 mM ammonium acetate buffer (pH 3, adjusted with acetic acid). The following gradient profile was used at a flow rate of 0.4 mL/min: 100% B for 0–1.5 min, decreasing to 92% at 3.5 min, decreasing to 90% at 7 min, decreasing to 78% at 10 min, decreasing to 65% at 11.5 min, decreasing to 2% B at 12 min, holding for 2 min, increasing to 100% at 15.5 min, and finally holding for 4.5 min to re-equilibrate the column. A mixture of 25 μL of serum and 15 μL of isotopically labeled standards (ca. 100 µM) was prepared and equilibrated on a shaker at room temperature for 30 min. Protein precipitation and analyte extraction were achieved by adding 95 μL of methanol to the samples, followed by shaking for 3 min and centrifugation (15 min, 4 °C, 11,337× *g*; centrifuge 5424 R, Eppendorf AG, Hamburg, Germany). The resulting supernatant was transferred to an autosampler vial and subjected to UHPLC-MS/MS analysis (injection volume 1 µL).

### 2.8. Quantification of hBD-1 Release by ELISA Assay

The photometric quantification of human beta-defensin-1 (hBD-1) in urine, serum, and EBC samples was performed using the BD-1 Mini ABTS ELISA Development Kits from PrepoTech (Hamburg, Germany), as recommended by the manufacturer. The absorbance was measured after 7–25 min of incubation by a microplate reader set at 405 nm and with 650 nm as wavelength correction.

### 2.9. Broth Microdilution Assay

The well-established, benign laboratory strain *Escherichia coli* (*E. coli*) K12 and *E. coli* CFT073, a mesophilic human pathogen originally isolated from the blood and urine of a woman with acute pyelonephritis, were purchased from Leibniz Institute DSMZ-German Collection of Microorganisms and Cell Cultures GmbH (Braunschweig, Germany). *E. coli* AM, isolated from a urine sample of an ambulant patient suffering from UTI, was kindly provided by the Dept. of Hygiene and Microbiology, University Medical Center Freiburg, Germany. Stock cultures for each strain were prepared from a 10 mL overnight culture in LB broth containing 10 g/L tryptone, 5 g/L yeast extract, and 10 g/L sodium chloride (18 h, 37 °C, 150 rpm) with 25% glycerol and stored as aliquots at −80 °C. The microdilution method was adapted from the Clinical Laboratory Standards Institute (CLSI) to test the antibacterial capacity of the urine samples (CLSI, 2015). A bacteria suspension of 1 × 10^8^ colony forming units (CFU)/mL was diluted in freshly prepared double-concentrated Mueller Hinton (Sigma-Aldrich, Taufkirchen, Germany) cation-adjusted broth to reach a test inoculum of approximately 1 × 10^5^ CFU/mL, which is defined as a diagnostic criterion for urinary tract infection. A quantity of 100 µL of the bacterial suspension was added to the wells of a 96-well U-shaped bottom plate. As positive control, ampicillin (20–2.5 µg/mL, Sigma-Aldrich, Taufkirchen, Germany) was used. A quantity of 100 µL of the antimicrobial agent or 100 µL of the centrifuged (4500× *g*, 10 min) urine samples was added in duplicate to the bacterial suspension in the wells. The plate was sealed and incubated at 37 °C for 7 h without shaking. Absorbance was measured at 600 nm immediately after resuspension and the values were subtracted from a negative control (water and medium). The minimal inhibitory concentration (MIC) for the antibiotics was determined as the lowest concentration that could still inhibit the visible and spectrophotometric detectable growth of the bacteria.

### 2.10. Statistical Analysis

The significance of the difference in OTU abundances between verum and control treatment was determined using a Wilcoxon signed-rank test with the resulting *p*-values being controlled for multiple testing via the Benjamini–Hochberg procedure.

To describe the α-diversity of each sample, Berger–Parker, bootstrap, Chao, inverse Simpson, and Shannon indices were calculated based on the OTU abundances of each sample. To measure β-diversity, principal coordinate analysis (PCoA) and non-metric multidimensional scaling (NMDS) of the thetaYC distance matrix among all samples calculated based on their 97% OTU composition and abundances were conducted. To determine significant differences in β-diversity, analysis of molecular variance (AMOVA) and homogeneity of molecular variance (HOMOVA) tests were applied.

To evaluate the change in hBD-1 expression or antibacterial activity upon intervention, a paired Student’s *t*-test was conducted using the GraphPad Prism 6.0 software (LaJolla, CA, USA). Here, *p*-values ≤ 0.05 (*) and ≤0.01 (**) were considered statistically significant and highly statistically significant, respectively.

For the Spearman correlation analysis, the OTU and metabolite abundances were log2-transformed. Species OTU counts were aggregated to genus level counts using the tax_glom function of the phyloseq R package. To determine the fold changes of the metabolites and OTUs caused by the verum intervention, the differences in the log2-transformed abundances before and after intervention were calculated for all metabolites and OTUs. The Spearman correlation was then calculated between the difference of metabolites and the difference of OTUs. Only those OTUs were included in this analysis that were present for both intervention and control. Additionally, only those subjects were included for which both metabolite and microbiome information was available. For the subgroup of individuals with a >25% upregulated serum PGE_2_, this resulted in information on 61 genera, 35 hydrophilic metabolites, and 8 SCFAs acquired from the same 10 subjects. For the subgroup of individuals with a >25% downregulated serum PGE_2_, this resulted in information on 56 genera and 35 hydrophilic metabolites for 5 subjects, which could be used for the correlation analysis between hydrophilic metabolites and the microbiome. For this subgroup, this also resulted in information on 54 genera and 8 SCFAs for 4 subjects, which could be used for the correlation analysis between SCFAs and the microbiome. For this subgroup, all subjects and all OTUs included for the correlation analysis between SCFAs and the microbiome were also present for the correlation analysis between hydrophilic metabolites and the microbiome.

## 3. Results

### 3.1. Microbiome Sequence Data

The final dataset after quality control consisted of 10,654,860 sequences from 116 samples (four stool samples each from 29 subjects) with an average length of approximately 450 base pairs, which corresponded to the length of the V3–V4 region (approximately 430 base pairs) [24]. From the initial dataset, a total of 5,922,871 reads were removed in quality filtering, mainly because sequence length was not sufficient. In total, this corresponded to approximately 50,000 reads filtered out per sample.

The mean ± SD bacterial OTU was 91,852 ± 38,244, with a maximum of 220,543 counts and a minimum of 34,894 counts per sample.

### 3.2. Microbial Composition

**Figure 1:**
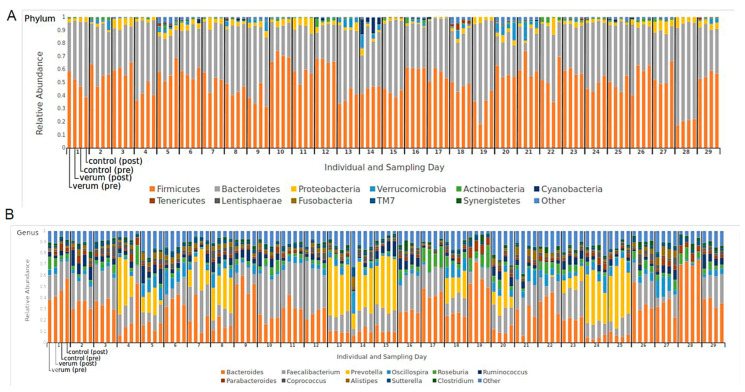
Microbial composition. Fractions of phyla (**A**) and genera (**B**) for each sample; *n* = 29 test subjects.

*Firmicutes* and *Bacteroidetes* were the two major phyla of bacteria found in the stool samples of the subjects (Figure 1A). A total of 55 families were identified. Seven families (*Ruminococcaceae*, *Bacteroidaceae*, *Lachnospiraceae*, *Prevotellaceae*, *Rikenellaceae*, *Porphyromonadaceae*, *Alcaligenaceae*) comprised >90% of sequences. The core set of typical bacterial families found in the intestines of healthy humans (*Bacteroidaceae*, *Clostridiaceae*, *Prevotellaceae*, *Eubacteriaceae*, *Ruminococcaceae*, *Bifidobacteriaceae*, *Lactobacillaceae*, *Enterobacteriaceae*) [33] was present in all samples.

At the genus level, it could be seen that all four groups were dominated by *Bacteroides* (23.06–24.46%), followed by *Faecalibacteria* (1.41–18.04%) and *Prevotella* (8.02–10.73%) (Figure 1B). A total of 101 genera were classified, with 12 representing >90% of total sequences (*Bacteroides, Faecalibacterium, Prevotella, Oscillospira, Roseburia, Ruminococcus, Parabacteroides, Coprococcus, Alistipes, Sutterella, Clostridium, Akkermansia*) and 70 representing <1%. No significant differences were found in the means of bacteria phyla and genera in the pre-to-post analysis for verum and control intervention. In some individuals, profound changes in microbiome composition could be seen during the study, however.

**Figure 2:**
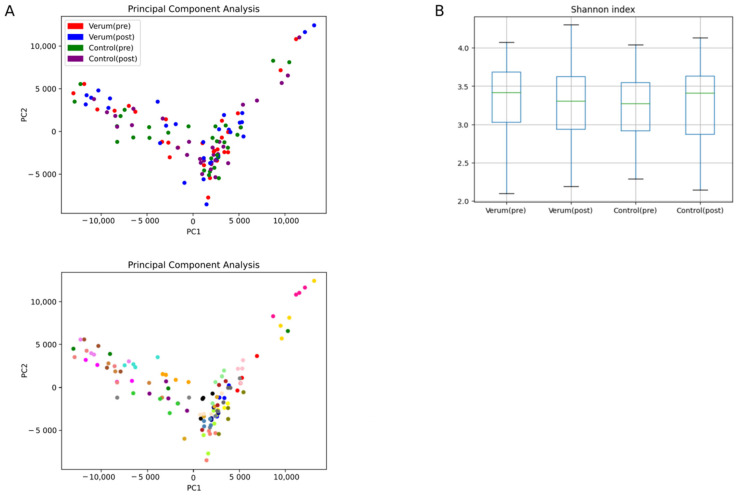
Microbial diversity analysis. Principal component analysis (**A**) using the k-means clustering algorithm (above colored by sampling day, below colored by individual), and boxplot of Shannon index as alpha diversity metric (**B**); *n* = 29 test subjects.

Using PCA, we found that samples were clustered by individuals rather than by time of sampling, which could suggest stability of the individual microbiome throughout intervention and high inter-individual variation in microbiome composition (Figure 2A). Walker et al. [34] also observed this in a clinical trial of 14 overweight men on different controlled diets containing indigestible starch or non-starch polysaccharides. They suggested that this could be associated with different inter-individual responses of the gut microbiota to dietary changes [34]. There were also no significant differences between baseline samples, which could be indicative of the absence of a carry-over effect between the two intervention phases.

**Figure 3:**
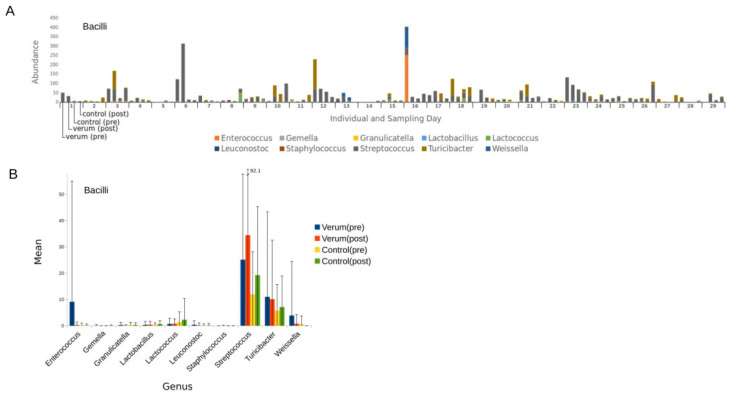
Abundance of bacteria from the class Bacilli. Absolute abundance per individual and sampling day of bacterial genera (**A**), and for each genus the mean and standard deviation per sampling day (**B**).

**Figure 4:**
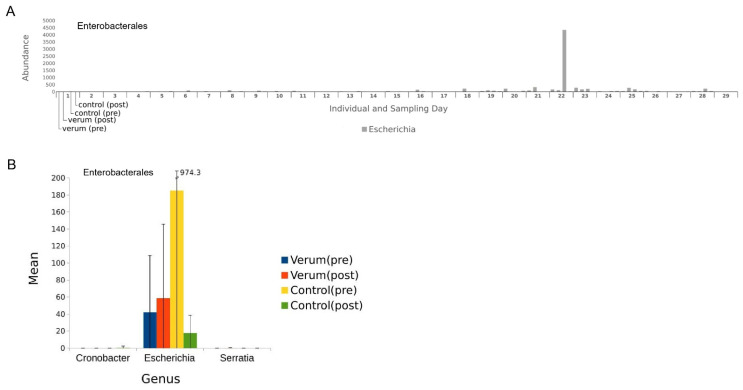
Abundance of bacteria from the order Enterobacterales. Absolute abundance per individual and sampling day of bacterial genera (**A**), and for each genus the mean and standard deviation per sampling day (**B**).

By further inspecting potentially uropathogenic bacteria such as members from the class of Bacilli and the order of Enterobacterales, we found some big differences in absolute abundance at an individual level, especially in the genera *Streptococcus*, *Turicibacter* (Figure 3A), and *Escherichia* (Figure 4A). Grouped by sampling day, there were again no significant changes in pre-to-post analysis for verum and control intervention in these genera, mainly because of a high SD, as seen in Figure 3B and Figure 4B.

### 3.3. Bacterial Diversity Is Not Affected by Nasturtium Intervention

In addition to the quantitative composition, the diversity of the samples was investigated. The results suggest that the bacterial microbiome of each subject remained relatively stable, although there were again large differences in diversity between individuals. Different alpha diversity metrics—observed OTUs, Shannon, Berger–Parker, bootstrap, Chao, and inverse Simpson indices—were used as measures of richness (biodiversity) and evenness (equitability) [35]. Nasturtium intervention did not significantly affect bacterial alpha diversity measures, which are representatively shown in Figure 2B for the Shannon index. For beta diversity, NMDS of thetaYC (Yue and Clayton theta similarity coefficient) distances between samples based on their 97% OTU composition and abundances showed that bacterial communities were also not significantly affected by nasturtium intervention (Supplementary Figure S1). This is in agreement with non-significant AMOVA (*p*-value = 1).

### 3.4. High-Level Phenotypes

After phylogenic imputation with PICRUSt [27], high-level phenotypes were determined, and some KEGG level-three modules were predicted using BugBase [29]. As seen in Supplementary Figure S2, treatment did not have a significant effect on these phenotypes.

### 3.5. Correlation of Gut Microbiome and Serum Metabolome

It has been shown that the composition of the diet and the gut microbiome can strongly influence the level of metabolites in the human serum [36,37]. We analyzed SCFA and amino acid content in serum samples before and after both intervention phases. As shown in Supplementary Figure S3, neither amino acid level (Supplementary Figure S3A–G) nor mean SCFA level (Supplementary Figure S3H–J) in serum were affected by the nasturtium intervention.

**Figure 5:**
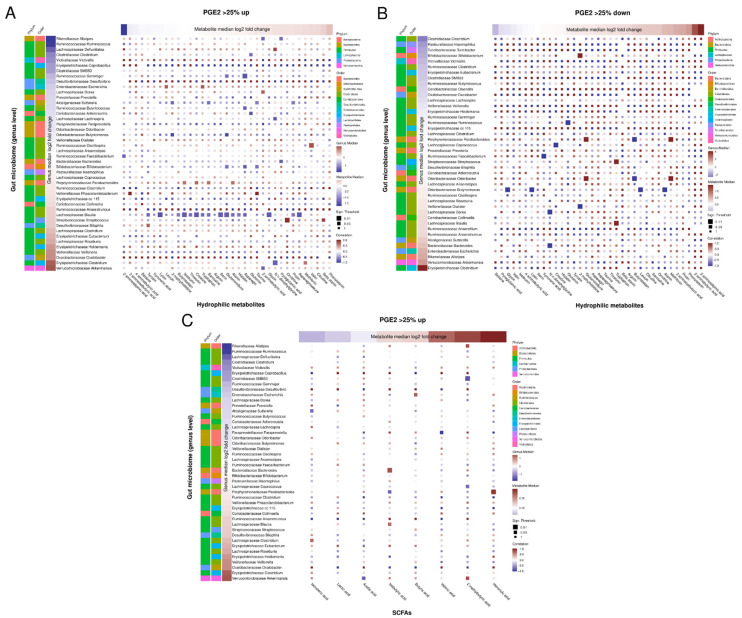
Spearman correlation analysis of the gut microbiome and metabolome. Spearman correlation analysis is conducted on the difference in the log2-fold changes of bacteria genera between pre vs. post treatment with verum and the log2-fold changes of metabolites between pre vs. post treatment with verum. Spearman correlation of the difference in bacteria genera and the difference in hydrophilic metabolites (**A**) for subjects with a >25% upregulated serum PGE_2_, for the difference in bacteria genera and the difference in hydrophilic metabolites for subjects with a >25% downregulated serum PGE_2_ (**B**), and for the difference in bacteria genera and the difference in SCFAs for subjects with >25% upregulated serum PGE_2_ (**C**). Correlation with an unadjusted *p*-value < 0.05 or 0.01 are emphasized by bigger squares in the correlation matrix. Positive correlation coefficients are displayed in red, negative ones in blue. To the left of the matrix, color-coded bars of the phylum and order of the respective genera are displayed. Additionally, to the left and above the correlation matrix, the median of the absolute difference in the log2-transformed abundance of bacteria genera (left) and metabolites (top) between verum and control is displayed as a color-coded bar, where blue corresponds to negative and red to positive values. No correlations were significant after FDR correction.

In our previous analysis, we found in 18 of 34 subjects a >25% increase in the primary study parameter, serum prostaglandin E_2_ (PGE_2_), upon nasturtium intervention (verum), while it was decreased by >25% in 9 subjects [19]. PGE_2_ is a multifunctional molecule that orchestrates a wide range of biological responses such as tissue homeostasis and inflammation, and it is one of the most abundant prostanoids in the human body [38,39]. We thus used the same subgroups of participants here to further investigate whether these large variations in PGE_2_ response upon nasturtium intervention are associated with a particular change in the composition of the gut microbiome, as previously proposed [40], or serum metabolome. To assess a possible relationship between changes in gut microbiota and circulating metabolites in these subgroups, we calculated the Spearman’s rank correlation coefficient between changes in OTUs and metabolite abundances caused by the verum treatment (Figure 5A,B). As an approximation for this difference in abundance (displayed in the left-side bar in Figure 5A,B) the median of the differences between the log2-transformed OTU abundances (pre vs. post treatment of verum) was used. After Benjamini–Hochberg correction of the computed *p*-values, Spearman’s test showed no significance (adjusted *p*-values > 0.05). Unadjusted *p*-values are shown below to indicate trends.

Among individuals with a >25% upregulated serum PGE_2,_ a lower abundance of five genera, namely *Ruminococcus*, *Clostridium (Clostridiaceae)*, *SMB53*, *Defluviitalea*, and *Gemmiger*, all belonging to the order Enterobacteriales, was detected after verum intervention (Figure 5A). Additionally, the genera *Alistipes*, *Victivallis*, and *Coprobacillus* were found with lower abundances in these subjects. Two genera of the family *Lachnospiraceae*, namely *Clostridium (Lachnospiraceae)* and *Roseburia*, and three genera of *Erysipelotrichaceae*, *Eubacterium*, *Holdemania*, and *Clostridium (Erysipelorichaceae),* were increased in this subgroup after nasturtium intervention. The genera *Veillonella*, *Oxalobacter*, and *Akkermansia* were also increased.

Analysis of the subgroup with a >25% decrease in serum PGE_2_ revealed a reduced abundance of the genera Clostridium (Clostridiaceae), Haemophilus, Turicibacter, Bifidobacterium, and Victivallis upon verum intervention in these subjects. Intervention-dependent increased abundance was observed in the genera Sutterella, Bacteroides, Escherichia, Alistipes, Akkermansia and Clostridium (Erysipelotrichaceae) (Figure 5B). Changes in the genus Bifidobacterium positively correlated here with changes in the hydrophilic serum metabolite 3-methylhistidine (*p* = 0.017) (Figure 5B).

In the subgroup with a >25% increase in PGE_2_ after the intervention, the changes in Alistipes for each subject were negatively correlated with the changes in 4-aminobutyric acid (*p* = 0.037) and pyroglutamic acid (*p* = 0.037), and positively correlated with the changes in 2-methylbutyric acid (*p* = 0.014) in serum. The changes in Defluviitalea positively correlated with the changes in N,N-dimethylglycine (*p* = 0.036), and the changes in *Clostridium (Clostridiaceae)* negatively correlated with the changes in glutamine (*p* = 0.043). The changes in *Gemmiger* negatively correlated with the changes in asparagine levels (*p* = 0.006), and *SMB53* negatively correlated with the changes in 2-methylbutyric acid (*p* = 0.006). The changes in *Clostridium (Erysipelotrichaceae)* were negatively correlated with the changes in serum content of N,N–dimethylglycine (*p* = 0.010) and positively correlated with changes in methionine (*p* = 0.050). Changes in Akkermansia positively correlated with changes in 2-methylbutyric acid (*p* = 0.022), and negatively correlated with changes in 2-aminobutyric acid (*p* = 0.048) and acetic acid (*p* = 0.022) (Figure 5A,B). In the subgroup with a >25% decrease in PGE_2_ after the intervention, the changes in the genus Bifidobacterium positively correlated with changes in the hydrophilic serum metabolite 3-methylhistidine (*p* = 0.017) (Figure 5B).

Correlations between bacterial genera and SCFAs in the subgroup with a >25% increase serum PGE_2_ are shown in Figure 5C. However, after Benjamini–Hochberg correction, all adjusted *p*-values were above 0.05. The lack of significance may be partly explained by the low sample number in both subgroups. No significant correlations were observed between changes in microbiota and changes in SCFAs for the subgroup with a >25% decrease in serum PGE_2_ (not shown).

We also compared the bacteria abundance between both subgroups; see Supplementary Figure S4A. Sutterella was found to be decreased in the subgroup with a >25% increase in PGE_2_, while it was increased in the subgroup with a >25% decrease in PGE_2_ (*p* = 0.0127 for Welch’s t-test, unadjusted). Eubacterium was increased in the subgroup with a >25% increase in PGE_2,_ while it was decreased in the subgroup with a >25% decrease in PGE_2_ (*p* = 0.0344 for Welch’s t-test, unadjusted).

However, for the comparison of serum metabolites between both subgroups (Supplementary Figure S4B), using Welch’s t-test no significant changes (*p* < 0.05) could be detected.

### 3.6. Nasturtium Intervention Increases hBD-1 in Urine and EBC

**Figure 6:**
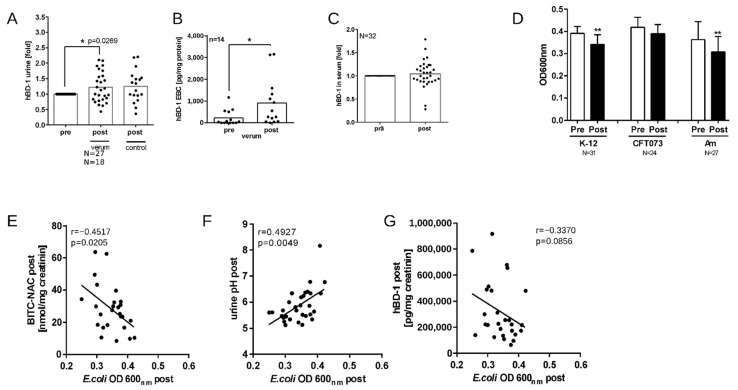
Antimicrobial effects of nasturtium. Quantification of hBD-1 in the samples before (pre) and after (post) nasturtium intervention was conducted via ELISA in urine (**A**), exhaled breath condensate (**B**), and serum (**C**). Each dot represents results from one subject, tested in duplicate. The line represents the mean. * *p* < 0.05 and ** *p* < 0.01. Urine samples were incubated for 12 h at 37 °C with 10^5^ CFU/mL of one of the three *E. coli* cultures. Antibacterial activity was measured at OD 600 nm as an indicator for bacterial growth (**D**). Correlations for *E. coli* bacterial growth and post-intervention BITC-NAC concentration (*n* = 26) (**E**), pH (*n* = 31) (**F**), and hBD-1 concentration (*n* = 27) (**G**) were calculated using Pearson (**F**) and Spearman (**E**,**G**) analyses. Statistical significance was described as *p* < 0.05 and *p* < 0.01.

Beta defensins are small cationic antimicrobial peptides, which are expressed in epithelial cells of the respiratory [41] and urinary tracts [42]. They kill or inhibit the growth of bacteria through a multiplicity of antimicrobial mechanisms, which include direct membrane disruption, inhibition of bacterial cell wall synthesis, or neutralizing secreted toxins [43]. While hBD-1 and hBD-2 are active preferably against Gram-negative bacteria [44], intervention of BITC-containing nasturtium significantly increased the hBD-1 level in urine (Figure 6A) and EBC (Figure 6B), but not in serum samples (Figure 6C).

### 3.7. Nasturtium Intervention Increases the Antibacterial Activity of Urine Samples

In order to investigate whether BITC-containing nasturtium could improve the antibacterial potential of urine, we used the collected spot urine samples from subjects before and after verum, and tested them against *E. coli* bacteria. Pre-to-post analysis showed that verum intervention significantly inhibited the growth of all three *E. coli* cultures, albeit this effect was weak (Figure 6D). Interestingly, the urinary content of BITC-NAC metabolite positively correlated with *E. coli* inhibition (r = 0.4517, *p* = 0.0205; Figure 6E). The correlation between the hBD-1 level in urine and bacterial inhibition was insignificant (Figure 6G), but there was also a significant positive correlation between the urinary pH and *E. coli* growth (r = 0.4927, *p* = 0.0049; Figure 6F).

## 4. Discussion

The primary advantage of using plant-based antimicrobials is that they can be administered without exhibiting the side effects often associated with the use of synthetic chemicals. In addition, antimicrobial resistance to their bioactive constituents is negligible, due to the multiple mechanisms of the constituents’ action potentially preventing selection of resistant bacteria strains [2,45]. On the other hand, the question of in vivo efficacy arises; in most cases this has yet to be proven. We could demonstrate here that no relevant perturbations to the gut microbiome in terms of evident changes in species richness or diversity could be detected upon nasturtium intervention, neither in the BITC-containing group nor the control without preformed BITC. In addition, the level of SCFA, as well as different amino acids in serum of subjects, demonstrated high stability during the whole course of the intervention. Considering the recommended use of nasturtium against infections of the upper respiratory tract and urinary tract [6,9], this could be advantageous. In contrast to our results, in rats exposed to broccoli, which contains the health-promoting ITC sulforaphane, compositional changes in the gut microbiota have been reported [46]. Another human study reported that sulfate-reducing bacteria were present in significantly lower levels in six of nine subjects after consuming *Brassica* vegetables over a two-week period [47]. Critically, however, bacteria of the genera *Bilophila* and *Desulfovibrio* were represented in these samples by an average of 0.049% and 0.033% of the total population, respectively. To our knowledge, in this study no error rate using mock community data was given, which would have been useful for assessing the reliability of these findings. Using a well-defined bacteria mock community in our study, we determined an error rate of 0.73%, which is in the range reported by others [48]. Thus, here we did not evaluate bacterial communities having an abundance below one percent. As with the present study, the usual eating behavior of the subjects was maintained in their study, and supplemented with the investigated plant. Subjects were only instructed to avoid GSL/ITC-containing foods during wash-out and intervention. More detailed data have been reported by [49] in a completely controlled 18-day human feeding study (N = 18). This was conducted with 200 g cooked broccoli and 20 g of raw daikon radish as the source of myrosinase per day. The authors found the relative abundance of Firmicutes significantly decreased, while both Bacteroidetes and the genus *Bacteroides* increased [49]. Subjects consumed all their meals on site, which might have been a critical advantage in avoiding microbiome changes due to individual food preferences. This could also explain their clear findings even at the phylum level. The broccoli used in all these mentioned studies mainly contains glucoraphanine, which is degraded to the aliphatic ITC sulforaphane [50]; nasturtium predominantly contains the aromatic structured BITC. For aliphatic ITC, a weaker antimicrobial activity has been reported in vitro compared to the aromatic ITCs [51,52]. Thus, the higher total intake in GLS as compared to our study could possibly have contributed to more pronounced effects on the gut microbiome despite a smaller study size.

Even though we could not find a statistically significant impact of nasturtium intervention on the mean abundance level and diversity of gut microbiota or serum metabolites, we observed great differences between individuals. As previously reported, nasturtium intervention led to elevated PGE_2_ serum levels of >25% in one part of the study participants, but decreased PGE_2_ by >25% in others [19]. Thus, here we performed a subgroup analysis to determine whether the change in PGE_2_ level upon intervention could be linked with some change in gut microbiota. Even though the effect was not statistically significant, we could identify several bacterial genera that changed in opposite directions in the two subgroups following the intervention. This was also true for some serum metabolites, and this observation should be investigated more closely in the future.

Some studies support the hypothesis that uropathogenic bacteria responsible for UTIs originate from the intestine [53,54,55]. Here, *E. coli* are the most common uropathogens found in patients with UTIs [54], which are then also often found in patients’ intestines [53]. Nowadays, the interaction between the gut microbiome and the UTI is intensively investigated as the “gut-UTI axis” [55]. Magruder et al. [55] found, for example, that antibiotic treatment altering the composition of the microbiome led to increases in the prevalence of the uropathogenic bacteria *Escherichia* or *Enterococcus*. Analysis of the number of species from the family *Enterobacteriaceae,* to which *E. coli* belongs, revealed no relevant changes upon intervention in the present study. This bacterial family was present in 28 of 29 subjects. However, one must keep in mind that the present study was conducted with healthy subjects, and we did not record whether the subjects had a history of UTI. Further, we performed V3–V4 sequencing of the microbiome, which limits species identification, and this could be considered here as a disadvantage, as well. In the future, patients actually suffering from UTI or having a history of UTI could be screened for the presence of certain uropathogenic bacteria in their gut microbiome, and then further considered for another intervention study on nasturtium.

For spot urine samples from BITC-containing nasturtium-treated subjects, a significant, albeit weak, growth inhibitory effect against *E. coli* bacteria was found. BITC-metabolite concentration in subjects’ urine then highly correlated with the observed antibacterial activity. Additionally, hBD-1, a quick-acting, anti-infective peptide in human host defense, was significantly upregulated upon nasturtium intervention. To date, there is little literature on the bioactivity of the ITC-NAC conjugates in general and, to our knowledge, none on the antimicrobial activity of the metabolite in particular. Preliminary evidence from inhibition experiments with cancer cells suggests here that the NAC conjugates are also active at a comparable potency [56]. This might be due to their pH-dependent dissociation capacity back into free ITC [57]. So far, several in vitro studies have shown sensitivity of both Gram-negative and Gram-positive bacteria to the antimicrobial properties of BITC [8,58]. BITC was effective against species of *Escherichia* and *Klebsiella*, as well as *Bacillus*, *Listeria*, *Salmonella*, *Serratia*, and *Staphylococcus* [58]. The concentration of applied BITC was, however, relatively high, which might provide a good explanation for the discrepancy between our results and these in vitro reports. For our experiments, only spontaneous urine samples were available instead of samples containing maximal expected BITC-metabolite levels. These urine samples then contained 41.3 ± 40.79 nM BITC-NAC. However, it is well known that the concentration of the ITC metabolite can reach up to several mM in the urine [59,60]. Platz et al. [59] demonstrated, for example, that upon a single consumption of 10 g fresh nasturtium juice, the urinary excretion of BITC-NAC peaked between 4 and 6 h at 830–2063 µM. Thus, our results are in principle useful to confirm the antimicrobial potential expected from ingested nasturtium in humans, but cannot provide information on the true antibacterial activity in urine.

In the human body, the main route of reactive, lipophile ITCs is their absorption in the small intestine and subsequent excretion via the urine as mercapturic acid pathway metabolites [61,62,63]. In animal experiments, only a minor part of <10% BITC/BITC-conjugate has been detected to be excreted via feces [64,65]. The intact hydrophilic and biologically inactive GLSs are expected to transit to the large intestine. There, some bacteria strains with *β*-thioglucosidase activity were shown to be involved in their further processing into the active ITC [66]. Interestingly, (a) other intestinal bacteria, e.g., *Bifidobacteria*, seem to rather metabolize GLSs to their corresponding inactive nitriles or amines [67,68]; and (b) results of one study on gnotobiotic rats even suggest that the human microflora may result in a lower net absorption of ITCs by the distal digestive tract due to breakdown to final products other than ITCs. Thus, while ITCs can reach the bladder at very high concentrations, much lower concentrations could be expected to reach the large intestine, which might help in protecting the gut microbiome from the antimicrobial properties of the ITC.

In future studies, establishment of a baseline gut microbiome, generated from at least three different sampling points before intervention, could help account for inter-individual variations and for the dynamic nature of the microbiome. This procedure has been suggested earlier [69] and could help to increase the sensitivity in the assessment of the effect of dietary intervention. The two control measurements carried out in the present study may be considered a limitation.

Another limitation of our study is that it was only conducted with women, which limits the transferability of the results. Women are more likely to experience UTIs than men [70]; furthermore, they differ in their innate and adaptive immune responses from men [71]. Results regarding sex differences in microbial taxa are inconsistent so far [72].

## 5. Conclusions

The effect of nasturtium on diversity and species richness of the bacterial gut microbiome was investigated here in the context of a secondary parameter analysis in healthy female participants. Our analyses did not reveal significant evidence for changes in the microbiome induced by BITC added to the individual diet. However, statistically significant antimicrobial effects of the intervention were found in breath condensate and urine. Furthermore, specific correlations between serum PGE_2_ and certain gut bacteria or serum metabolites were identified, which need to be validated in further studies.

## Data Availability

Galaxy Workflow for processing microbiome (16S rRNA) sequence data is accessible via https://usegalaxy.eu/u/spp/w/16s-rrna-gg-workflow (accessed on 17 May 2023).

## Supplementary Materials

The following supporting information can be downloaded at: https://www.mdpi.com/article/10.3390/nu16030373/s1, Figure S1: NMDS plot of thetaYC distances for three dimensions; Figure S2: Relative abundance of high-level phenotypes (*n* = 29 subjects) using BugBase web application; Figure S3: Metabolome analysis using QTRAP; Figure S4: Correlation of bacteria abundance between subgroups.
