# Correction: Pfäffle et al. A 14-Day Double-Blind, Randomized, Controlled Crossover Intervention Study with Anti-Bacterial Benzyl Isothiocyanate from Nasturtium (*Tropaeolum majus*) on Human Gut Microbiome and Host Defense. *Nutrients* 2024, *16*, 373

**DOI:** 10.3390/nu17081367

**Published:** 2025-04-17

**Authors:** Simon P. Pfäffle, Corinna Herz, Eva Brombacher, Michele Proietti, Michael Gigl, Christoph K. Hofstetter, Verena K. Mittermeier-Kleßinger, Sophie Claßen, Hoai T. T. Tran, Dhairya Rajguru, Corinna Dawid, Clemens Kreutz, Stefan Günther, Evelyn Lamy

**Affiliations:** 1 Molecular Preventive Medicine, University Medical Center and Faculty of Medicine, University of Freiburg, Engesserstrasse 4, D-79108 Freiburg, Germany; 2 Institute of Pharmaceutical Sciences, Faculty of Chemistry and Pharmacy, University of Freiburg, Hermann-Herder-Strasse 9, D-79104 Freiburg, Germany; 3 Institute of Medical Biometry and Statistics, University Medical Center and Faculty of Medicine, University of Freiburg, Stefan-Meier-Str. 26, D-79104 Freiburg, Germany; 4 Faculty of Biology, University of Freiburg, Schänzlestr. 1, D-79104 Freiburg, Germany; 5 Spemann Graduate School of Biology and Medicine (SGBM), University of Freiburg, Albertstr. 19A, D-79104 Freiburg, Germany; 6 Centre for Integrative Biological Signaling Studies (CIBSS), University of Freiburg, Schänzlestr. 18, D-79104 Freiburg, Germany; 7 Center for Chronic Immunodeficiency (CCI), Microbiome Core Facility, Breisacher Strasse 115, D-79106 Freiburg, Germany; 8 Chair of Food Chemistry and Molecular Sensory Science, TUM School of Life Sciences, Technical University of Munich, Lise-Meitner-Strasse 34, D-85354 Freising, Germany; 9 Bavarian Center for Biomolecular Mass Spectrometry, TUM School of Life Sciences, Technical University of Munich, Gregor-Mendel-Strasse 4, D-85354 Freising, Germany; 10 Institute of Molecular Medicine and Cell Research, Faculty of Medicine, University of Freiburg, D-79104 Freiburg, Germany

## Addition of an Author

Dhairya Rajguru was not included as an author in the original publication [1]. The corrected Author Contributions statement appears here:

**Author Contributions:** Conceptualization, E.L.; methodology, S.P.P., E.B., M.P., M.G., C.H., V.K.M.-K., C.D., S.C., H.T.T.T., C.K., C.K.H. and S.G.; formal analysis, C.H., H.T.T.T., D.R., S.C. and E.L.; investigation, C.H., E.B., H.T.T.T., S.C. and V.K.M.-K.; resources, C.D., S.G. and E.L.; writing—original draft preparation, E.L.; writing—review and editing, S.P.P., C.H. and S.G. All authors have read and agreed to the published version of the manuscript.

## Additional Affiliation

In the published publication, there was no affiliation information for Dhairya Rajguru. The newly added affiliation ten should include the Institute of Molecular Medicine and Cell Research, Faculty of Medicine, University of Freiburg, D-79104 Freiburg, Germany.

The authors state that the scientific conclusions are unaffected. This correction was approved by the Academic Editor. The original publication has also been updated.
