# Supplementary material for: A 14-Day Double-Blind, Randomized, Controlled Crossover Intervention Study with Anti-Bacterial Benzyl Isothiocyanate from Nasturtium (Tropaeolum majus) on Human Gut Microbiome and Host Defense
Source: Nutrients. 2024 Jan 26;16(3):373. doi: 10.3390/nu16030373 (PMC10857499; doi:10.3390/nu16030373)

Supplementary Figure S1: NMDS plot of thetaYC distances for three dimensions.

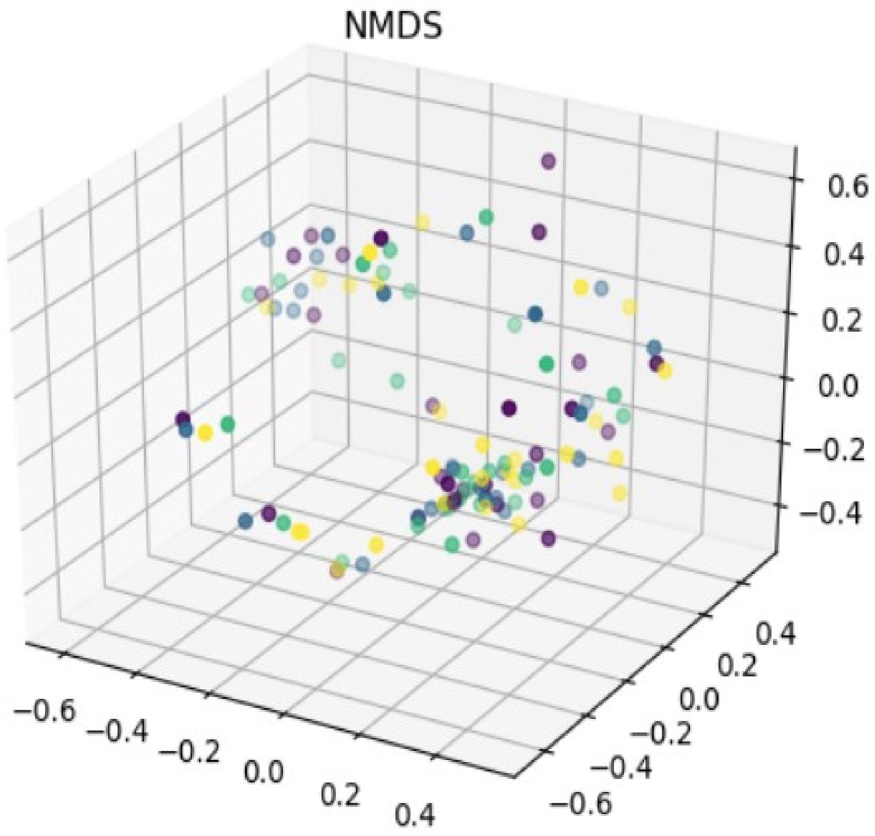

Supplementary Figure S2: Relative abundance of high-level phenotypes (*n* = 29 subjects) using BugBase web application.

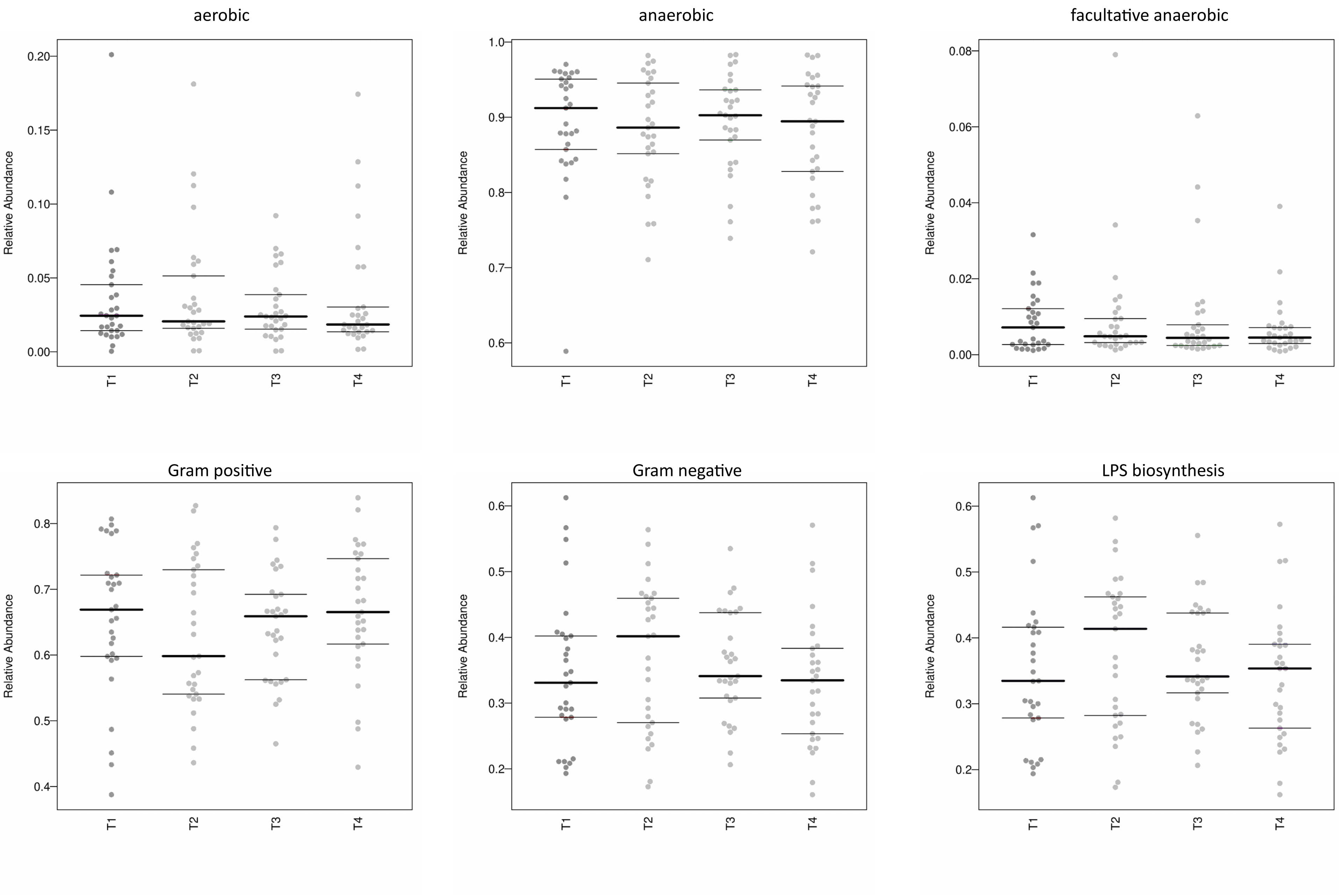

Supplementary Figure S3: Metabolome analysis using QTRAP.

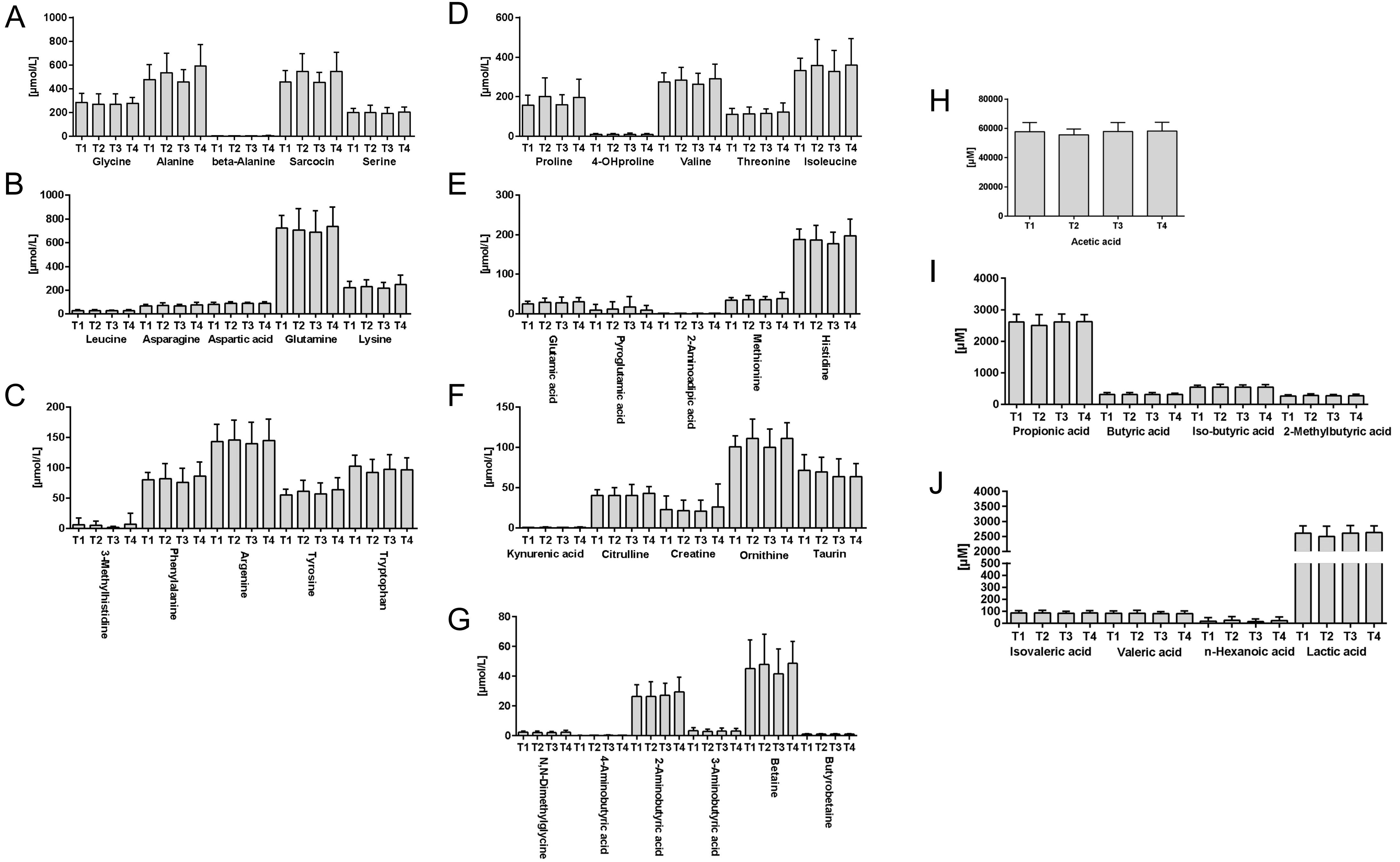

Supplementary Figure S4: Correlation of bacteria abundance between subgroups.

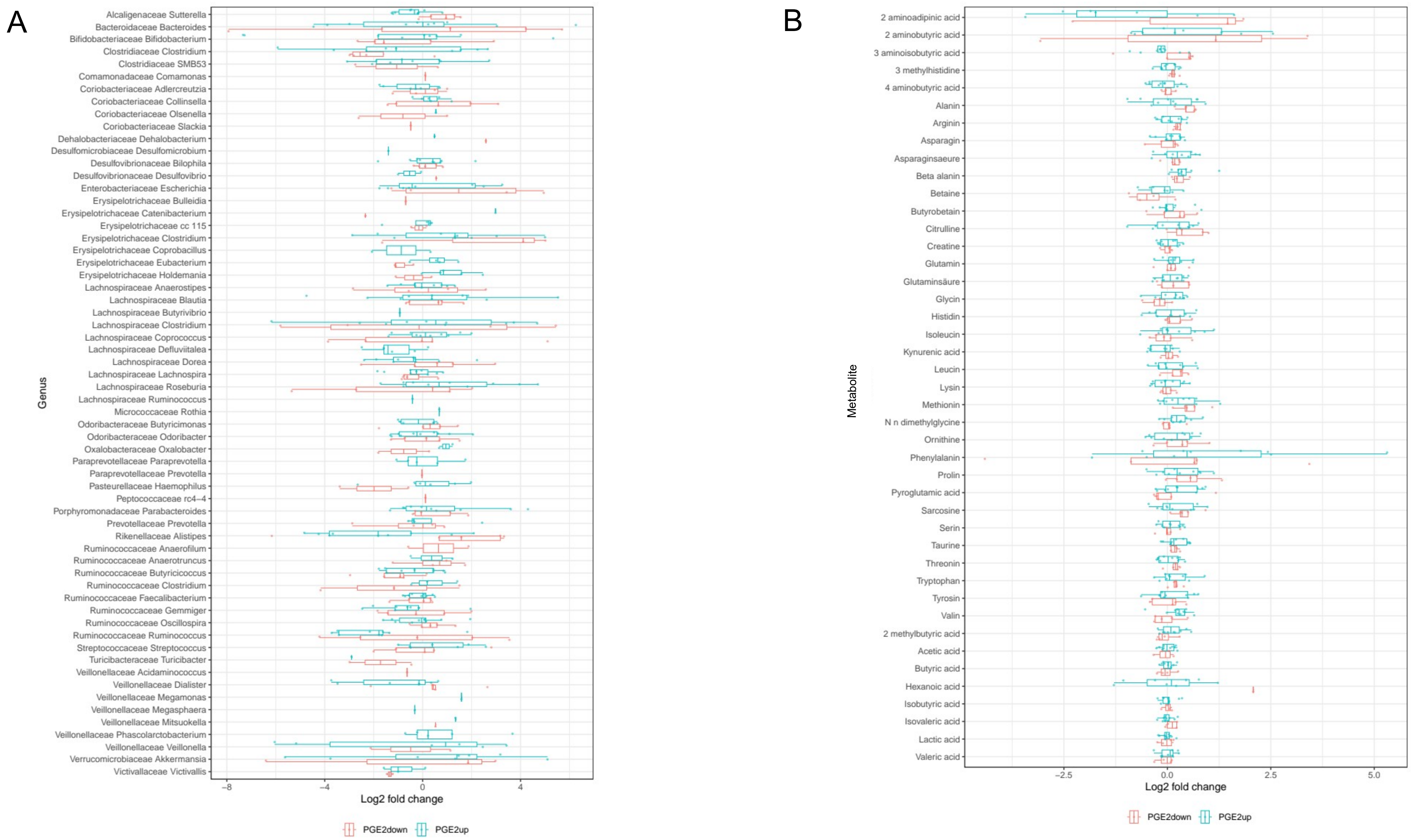

Supplement: Supplementary file 1 [file nutrients-16-00373-s001.zip › nutrients-2752294-supplementary.pdf]
